# What are the key factors influencing consumers’ preference and willingness to pay for meat products in Eastern DRC?

**DOI:** 10.1002/fsn3.813

**Published:** 2018-10-04

**Authors:** Patchimaporn Udomkun, John Ilukor, Jonathan Mockshell, Gaudiose Mujawamariya, Christopher Okafor, Renee Bullock, Nsharwasi Léon Nabahungu, Bernard Vanlauwe

**Affiliations:** ^1^ International Institute of Tropical Agriculture (IITA) Bujumbura Burundi; ^2^ World Bank Development Data Group‐Survey Unit Kampala Uganda; ^3^ International Center for Tropical Agriculture (CIAT) Cali Colombia; ^4^ Africa Rice Center (AfricaRice) Antsirabe Madagascar; ^5^ IITA Bukavu The Democratic Republic of Congo; ^6^ IITA Nairobi Kenya

**Keywords:** consumer's perception, meat products, quality attributes, socio‐demographic factor, willingness to pay

## Abstract

Dietary patterns for consumers among the elite and middle‐income classes in developing countries are shifting rapidly toward the consumption of more animal‐based products. Although this shift presents opportunities, there are significant market failures affecting their preferences and willingness to pay (WTP). This study used a multistage sample survey of 309 consumers from three different communities of Bukavu, Eastern DRC, to examine the effect of socioeconomic/socio‐demographic characteristics and quality attributes on consumers’ purchasing decisions and WTP for meat products. The results suggested that about 53% of the respondents were dissatisfied with meat products in the market due to their high price, low quantity, unhealthiness, and harmful effects. Older female respondents living in urban areas were more likely to purchase meat products. Their WTP was significantly determined by attributes such as color, in‐mouth texture, and availability. Nutrition, harmful effects, and availability of meat products are the important factors that influence purchasing decisions among higher income groups. Addressing these market failures could have an impact on the meat market, improving the nutrition of low‐income consumers and ensuring food safety standards in DRC and other developing countries with similar challenges.

## 1 INTRODUCTION

From the perspective of healthy nutrition and well‐being, meat is a good source of protein, minerals (iron, zinc, calcium), and vitamins (A, B12 and other B vitamins) (Pereira & Vicente, 2013; Randolph et al., 2007). As part of a nutrition transition (Popkin, Adair, & Ng, 2012) and livestock revolution (Delgado, 2003), growth of meat consumption in developing countries is likely to increase. According to FAO (2014), average annual consumption of meat in developed countries is 75.5 kg/inhabitant, while consumption of 33.9 kg/inhabitant is estimated in developing countries. Worldwide, levels of meat consumption are projected to increase by 72% in 2030 compared to the situation in 2000 (Fiala, 2008). In sub‐Saharan Africa, the demand for meat products is also growing rapidly, increasing by 140% between 2000 to 2030 (FAO, 2011).

The projected increase in meat consumption is a sign of a better future with regard to malnutrition levels among the poor in lower‐income countries who suffer from micronutrient deficiencies and mainly depend on high fiber and phytate plant‐based staples (Neumann et al., 2003). The impact of malnutrition is globally estimated to be as high as US$3.5 trillion per year or US$500 per individual (FAO, 2013). The costs are opportunity costs of economic growth foregone and lost investments in human capital resulting from infections, impaired child development, and mortality (Hoddinott, 2016). In the Democratic Republic of Congo (DRC), over 3.6 million children under five are affected by acute malnutrition annually and 2 million of them suffer from its most severe form (OCHA, 2016). This country is estimated to be losing more than a billion dollars a year to the effects of child undernutrition, which is equivalent 4.5 percent of GDP. Therefore, consumption of meat products could be one of the keys to reducing malnutrition costs in the DRC.

However, as argued by Randolph et al. (2007), the negative publicity on livestock and their products is driven by health and food safety concerns related to outbreaks of diseases like avian influenza and the continued debates on the association between the saturated fats and cholesterol found in animal food sources and chronic diseases like heart disease and cancer, contributing to consumer nervousness about meat products. Consumer nervousness affects their WTP, purchase, and consumption of meat products, thus exacerbating the malnutrition level and related costs in developing countries. Nevertheless, consumers’ choices are influenced by many factors that ultimately shape purchasing decisions. Font‐i‐Furnols and Guerrero (2014) identified consumers’ behavior as depending on interrelated factors that included psychological influences (willingness, risk, expectations, sociocultural factors, lifestyle, and values), sensory qualities (visual appearance, texture, flavor, and odor), and marketing factors (price, label, brand, and availability). In addition, Grunert, Bredahl, and Brunsø (2004) used the Total Food Quality model to analyze consumers’ perception and decision‐making in determining meat quality. The model showed that consumers form expectations about quality at the point of purchase, based on their own experience and informational cues available in the shopping environment.

These preferences are influenced not only by quality and consumer‐related factors but also by context, culture, and information (Kanerva, 2013; York & Gossards, 2004). Alemu, Olsen, Vedel, Pambo, and Owino (2017) showed that preferences in Kenya are also influenced by context and information in addition to product attributes. Van Wezemael, Verbeke, de Barcellos, Scholderer, and Perez‐Cueto (2010) also reported that European consumers considered label, brand, freshness, and leanness of beef as cues to indicate quality to purchase, whereas safety in Ghana and hygiene in Rwanda were purchasing attributes in purchasing meat products (Niyonzima et al., 2017; Owusu‐Sekyere, Owusu, & Jordaan, 2014). However, most of the studies on consumers’ preferences for meat products focus on developed countries (Tonsor et al., 2005; Reicks et al., 2011; Schumacher, Schroeder, & Tonsor, 2012; Zimmerman et al., 2012; Hung, de Kok, & Verbeke, 2016; Shan et al., 2017). Only a few studies focus on the African context where food quality and malnutrition remain huge challenges (Niyonzima et al., 2017; Owusu‐Sekyere et al., 2014). Increasing incomes in developing countries together with the inherent market failures makes it vital to understand the factors driving consumers’ meat consumption patterns and their WTP for such food products. Failure to understand the key determinants of consumers’ preferences could lead to further market failure and the consumption of unwholesome meat products (Mockshell, Ilukor, & Birner, 2014).

The overall objective of this study is to evaluate the preferences for meat and meat products and WTP among consumers in Eastern DRC. Specifically, this study aims at: (a) identifying consumer and household characteristics influencing consumer preferences and WTP; (b) examining consumers’ preferences for meat products; and (c) analyzing the effect of socio‐demographics and product attributes on purchasing decisions and WTP by using linear and ordered multinomial logistic regression models. The rest of the paper is structured as follows: Section 2 presents the structure of the meat market in the DRC, and Section 3 presents the materials and methods. The results are presented in Section 4 and discussed in Section 5; the paper presents the conclusions in Section 6.

## 2 THE MEAT MARKET IN THE DEMOCRATIC REPUBLIC OF CONGO

**Figure 1:**
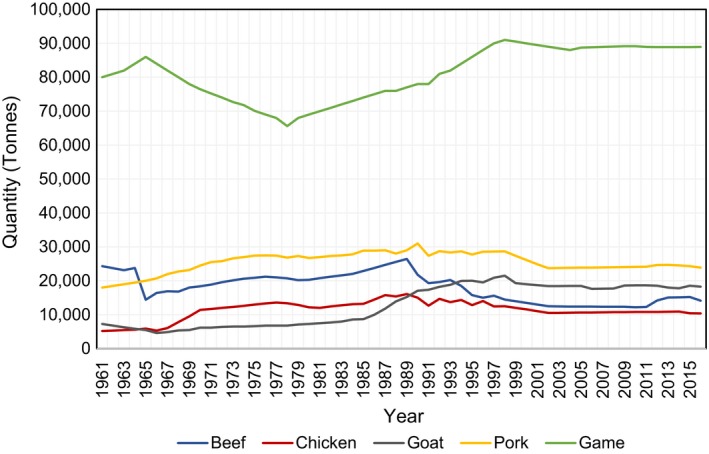
Meat production in the Democratic Republic of Congo by type of livestock. Source: FAOSTAT (2018)

The agricultural sector is an important sector in the economy of the DRC. Its accounts for 21% of GDP and employs about 70% of the population (KPMG, 2016). The proportion of livestock to the agricultural GDP is only 9%, and the livestock sector is largely undeveloped, with small numbers of cattle, pigs, goats, and chickens. The livestock population is estimated to be seven million, and 60% are goats, 15% pigs, 14% sheep, and 11% cattle (FAO, 2005). Livestock populations have suffered significantly since the civil war, when many farms were looted and the animals stolen. As an important source of dietary protein, consumption and sale of wild animals (“bushmeat”), including some primates, are widespread. This has been fueled partly by poor living conditions and the rise in the number of internally displaced people (IDPs) fleeing regional conflicts. As shown in Figure 1, wild meat is the most produced meat product in the DRC followed by pork and beef.

**Figure 2:**
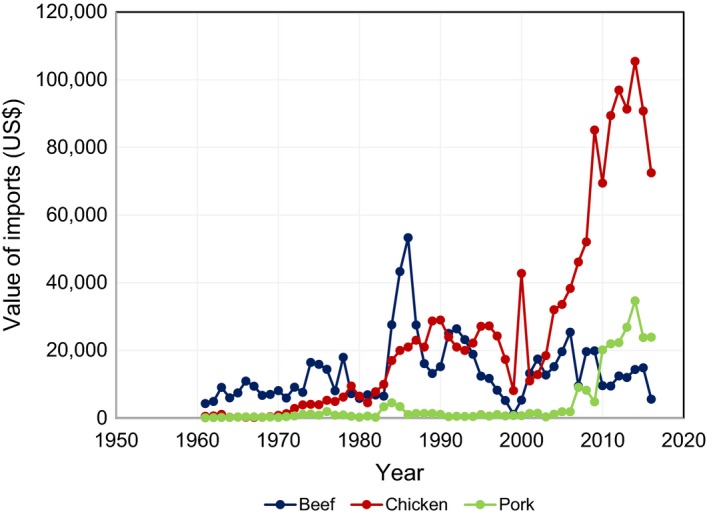
Value of meat imports (US$1,000) in the Democratic Republic of Congo between 1961 and 2016. Source: FAOSTAT (2018)

The consumption of meat is higher than the production of meat in DRC, so the country is a net importer of food products (Food and Agriculture Organization (FAO), 2005). The main exporters of meat to the DRC are South Africa, India, and European Union (EU) countries like the Netherlands that is the largest exporter of pork together with Belgium and Germany. As shown in Figure 2, the main imported meat is chicken followed by pork. Meat imports have generally declined from the 1980s to date. The decline in beef imports can be linked to the deliberate effort of government to promote cattle production through the rehabilitation of some cattle farms that were destroyed by the wars, particularly in Katanga Province and North and South Kivu (Goma and Masisi) in the Northeast bordering Rwanda and Burundi as well as the increased consumption and preference of game (Yamaguchi, 2015). Another contributory factor is increased concerns of meat quality especially contamination with salmonella, which is a threat to human health (Mahangaiko, Mabi, Bakana, & Nyonggombe, 2015). Makelele et al. (2015) in a study to assess microbial quality of meat sold by street vendors in Kisangani Province in DRC found that 90% of the samples had unsatisfactory microbiological quality due to *Salmonella* sp. (57.1%) and *Staphylococcus aureus* (50%).

In Eastern DRC, limited economic growth during the past few years has created an expanding middle‐ and high‐income population (Maass, Musale, Chiuri, Gassner, & Peters, 2012; Van Acker, 2005; Vlassenroot, Ntububa, & Raeymaekers, 2003). Subsequently, these consumers’ dietary patterns have changed swiftly toward higher levels of consumption of meat products, while those in the low‐income category continue to suffer extreme poverty and malnutrition (Kandala, Madungu, Emina, Nzita, & Cappuccio, 2011; Rossi, Hoerz, Thouvenot, Pastore, & Michael, 2006). Generally, beef, goat, pork, chicken, and rabbit are the meats most consumed in Eastern DRC. Some of them are produced in‐country, while others are obtained from neighboring countries, including Rwanda and Uganda. Butchers commonly sell in small or large quantities of cut meat, although goats, chickens, and rabbits are generally sold live and slaughtered at home. High‐ and middle‐income households purchase beef or goat meat but lower‐income households often choose smaller animals such as pigs, poultry, and rabbits, as their coping strategy (Maass et al., 2012). However, small animals are sold in the markets only when household needs arise, and the money raised is mostly invested in school fees (Zozo et al., 2012).

## 3 MATERIALS AND METHODS

### 3.1 Sampling and survey design

A consumer study survey was conducted between April and June 2017 in three communities (Ibanda, Bagira, and Kadutu) of Bukavu city Eastern DRC. A multistage random sampling procedure was used to select respondents for the interviews. In the first stage the three communities were purposively selected, based on the consumers’ different socioeconomic backgrounds. The list of Quarters (subunits) in the community was first obtained from the Provincial Inspection for Agriculture and Livestock (IPAPEL). Within each community, a list of Quarters was generated, and a representative proportion was randomly selected. The Quarters were Ndendere, Nyalukemba, and Panzi for Ibanda; Nkafu, Mosala, and Kasali for Kadutu; and Quarter A, Quarter B, Quarter C, and Quarter D for Bagira. Within each quarter, a list of households was generated and random samples of 309 were selected for interview based on the probability proportional to size (PPS) sampling approach. This PPS approach was used because the household population is not the same in each Quarter. Interviews were conducted with selected respondents face‐to‐face by trained enumerators using a semi‐structured questionnaire administered in Kiswahili, Mashi (local Congolese language), and French. To ensure that the respondents understood the concept, the enumerators were requested to explain the unfamiliar terms to the respondents, use illustrations, and test their understanding of key terms before administering the questionnaire.

The survey questionnaire was structured in three modules. The first module covered household composition and characteristics such as region of residence, gender, age, marital status, education level, occupation, and household size. The second module included questions on income, expenditure, and household decision‐making. Respondents were asked who was the main breadwinner in their household and who decides which food to purchase. In the third module, respondents were asked about their consumption and purchasing frequencies of meat products including beef, pork, goat, chicken, and rabbit. In the fourth module, respondents were asked how satisfied they were with the meat products and the factors influencing their purchasing decisions and WTP.

To evaluate the willingness of consumers to pay, the revealed preference method was applied. The method was chosen because data obtained from revealed preference methods more truthfully reflect preferences and choice in the real market when compared to stated preference methods (Howard & Allen, 2008). Respondents were given the average prices based on the different markets for each meat product, and then they were asked to score the influence of product attributes (nutrition, color, texture, smell, harmful effect, price, availability, and quantity) on their perception (no = 0, yes = 1). In addition, they were asked to rank the importance of these attributes on their purchasing decisions by using a five‐point Likert scale (not important/definitely would not pay = 1, least important/probably would not pay = 2, moderately important/might pay = 3, important/probably would pay = 4, and most important/definitely would pay = 5).

### 3.2 Data analysis

Data analysis was performed using R software (version 3.2.3, R Core, 2015). Basic statistics (means, standard deviation, and frequencies) were computed to describe the responses. Chi‐square (*χ* ^*2*^) and analysis of variance (ANOVA) were used to examine the differences in the responses. In order to fit linear regression assumptions for ANOVA, BoxCox power transformations were applied to the continuous variables; the transformed variables were analyzed using ANOVA, and the mean comparisons were done on the back‐transformed values (Box & CoX, 1964). Significantly different means were separated using least significant difference (LSD) with the appropriate error terms and a significance level at *p* < 0.05.

To investigate the factors determining purchasing decisions and WTP among respondents, a logistic regression analysis was performed following a generalized linear regression with *probit* link. When *Y* is the dependent or response variable as *Y* is dichotomous, the use of *probit* link, *f*(*Y*), leads to the transformation of the response into a continuous variable, *Y*. The link function then maps the (0, 1) range probabilities onto (−∞,+∞), the range of linear predictors (Agresti, 2002; Fox, 2008). We then have a *probit* model as:

$$Y=\Phi (\beta_{0}+X_{i}\beta_{i}+\varepsilon )$$

$$\Phi^{-1}\left(Y\right)=\beta_{0}+X_{i}\beta_{i}+\varepsilon$$

$$Y^{\prime }=\beta_{0}+X_{i}\beta_{i}+\varepsilon$$

The *probit* link function is given by Faraway (2006) as:

$$f\left(Y\right)=\Phi^{-1}\left(Y\right)$$

where *Φ* ^‐1^ is the inverse normal cumulative distribution function, such as *N* (0, 1) (Agresti, 2002).

And the regression equation becomes:

$$f\left(Y\right)=Y^{\prime }=\beta_{0}+X_{i}\beta_{i}+\varepsilon$$

The model parameters were estimated using the maximum‐likelihood method, with chi‐square test of significance (Dodge, 2008). The following vector of independent variables was considered for their socio‐demographic effects:

$$\begin{array}{cc} X_{i}= & \{\text{LIVING AREA, GENDER, AGE, MARRITAL STATUS, EDUCTION.}\\ & \;\text{EMPLOYMENT, HOUSEHOLD SIZE, CHILDREN, INCOME}\} \end{array}$$

These are standard socio‐demographic variables such as living area, gender, current age, marital status, education level, and employment status of household head, household size, and income. Table 1 describes the independent variables used in the linear regression model.

**Table 1:** Description of variables used in the model

Variable	Description
Living area	1 if rural, 0 otherwise
Gender	1 if female, 0 otherwise
Age	Years
Marital status	1 if married, 0 otherwise
Education level of household head	0 if none/primary school, 1 if junior/secondary 2 if college/university
Employment status of household head	1 if employed/work, 0 otherwise
Household size	Number of members in a household
Children	1 if having children in the household, 0 otherwise
Household annual income	Household income for last 12 months (USD)
Nutritious	1 if nutritious of products influences on purchasing decision/willingness to pay, 0 otherwise
Color	1 if color of products influences on purchasing decision/willingness to pay, 0 otherwise
Texture	1 if texture of products influences on purchasing decision/willingness to pay, 0 otherwise
Taste	1 if taste of products influences on purchasing decision/willingness to pay, 0 otherwise
Harmful effect	1 if harmful effect of products influences on purchasing decision/willingness to pay, 0 otherwise
Price	1 if price of products influences on purchasing decision/willingness to pay, 0 otherwise
Availability	1 if availability of products influences on purchasing decision/willingness to pay, 0 otherwise
Quantity	1 if quantity of products influences on purchasing decision/willingness to pay, 0 otherwise
Perception	1 if perception of products influences on purchasing decision/willingness to pay, 0 otherwise

The effects of product attributes (nutrition, color, texture, harmful effect, price, availability, and quantity) on consumers’ purchasing decisions and WTP were determined by performing an ordered multinomial logistic regression model, as the above dependent variables were nominal and polytomous, i.e. had more than two categories with an ordered structure (Engel, 1988; Menard, 2002).

When the following ordered probit model estimated using maximum‐likelihood (ML) method is considered, we have

$$Y_{n}^{*}=x_{n}^{\prime }\beta +\varepsilon_{n}$$

with $y_{n}^{}*$ is the unobserved dependent variable, $x_{n}^{\prime }$ is the vector of independent variables, and *β* is the vector of regression coefficient to estimate. The latent random variable $y_{n}^{*}$ for individuals *n* = 1,2,3…*N*, linearly depends on the independent variables *x*_*n*_ and *ε*_*n*_ is the error term. Therefore,

$$y_{n}=\left\{\begin{array}{c} 1\text{if}{y_{n}}^{*}\leq \beta_{1}\\ 2\text{if}\beta_{1}<{y_{n}}^{*}\leq \beta_{2}\\ 3\text{if}\beta_{2}<{y_{n}}^{*}\leq \beta_{3}\\ j\text{if}\beta_{j-1}<y_{n} \end{array}\right.$$

If the errors *ε*_*n*_ are logistically distributed, with distribution function $\Lambda \left(\varepsilon_{i}\right)=\frac{1}{1+e^{-\varepsilon_{i}}}$ produces an ordered logistic model given by Akshita, Ramyani, Sridevi, and Trishita (2013) as:

$$\text{logit}\left[Pr\left(y_{i}>J\right)\right]={\text{log}}_{e}\frac{Pr\left(y_{i}>J\right)}{Pr\left(y_{i}\leq J\right)}=-\alpha_{j}x_{i}^{\prime }\beta ..forJ=1,2,\ldots ,j$$

With regard to household income, the influence of product attributes on purchasing decisions and WTP was explained by the gg plots.

## 4 RESULTS

### 4.1 Consumer and household characteristics

Most of the respondents (86%) lived in urban areas, and the majority were female (56%) with an average age of 37 years (Table 2). The average household size was 6 persons, and the composition is characterized by 54% of children, 18% of the household head, 15% of the spouse. In this study, 87% had attained at least primary school, with an average of 8 years of formal education. Most of the respondents in Ibanda had completed higher education when compared to those in Kadutu and Bagira. The main occupation of respondents varied among communities. Self‐employed business/services (26%) was observed as a main occupation in Ibanda, whereas many respondents in Kadutu (20%) and Bagira (20%) were unemployed.

**Table 2:** Socio‐demographic characteristics of samples in Bukavu city, Eastern DRC

Variables	Community			Total	*χ* ^2^	*p*‐value
	Ibanda (*N* = 99)	Kadutu (*N* = 110)	Bagira (*N* = 100)			
Living area (%)						
Rural	0.0	0.9	1.1	0.6	57.06	<0.001*^**^
Urban	100.0	92.1	64.9	85.7		
Peri‐urban	0.0	7.0	34.0	13.7		
Sex (%)						
Male	48.0	38.3	44.6	43.6	2.15	0.341
Female	52.0	61.7	55.4	56.4		
Age (years)^a^	35.6 (12.6)	37.0 (15.8)	37.2 (11.2)	36.6 (13.5)		0.572
Household composition (%)						
Head	15.9	20.3	18.2	18.1	53.99	<0.001*^**^
Spouse	13.6	15.8	14.0	14.5		
Son/daughter	54.9	46.9	60.2	54.1		
Grandchild	1.6	1.9	3.1	2.2		
Hired worker	4.4	5.1	0.5	3.3		
Other (parent, brother/sister)	9.6	10.0	4.0	7.8		
Household size (number)^a^	6.4 (2.8)	6.1 (2.1)	6.2 (2.2)	6.2 (2.4)		0.411
Marital status of respondents (%)						
Never married	26.0	33.0	16.0	25.0	21.09	0.021*
Married living with spouse	66.0	48.7	72.3	62.3		
Married but spouse away	2.0	7.0	3.2	4.0		
Other (separated, divorced, widow/widower)	6.0	11.3	8.5	8.7		
Household education (years)^a^	8.8 (6.5)	8.0 (5.5)	7.9 (5.7)	8.2 (5.9)		0.027*
Education level of respondents (%)						
None	8.0	17.9	12.9	12.9	50.76	<0.001*^**^
Primary	10.0	25.9	17.2	17.7		
Secondary	24.0	40.2	44.1	36.1		
Graduate	27.0	8.0	15.1	16.7		
Bachelor	26.0	7.1	10.7	14.6		
Other (master, doctorate)	5.0	0.9	0.0	2.0		
Main occupation of respondents (%)						
Crop farming	1.0	3.6	4.3	3.0	32.51	<0.001*^**^
Self‐employed business/services	26.0	8.0	7.2	13.7		
Domestic work in own home	7.3	16.2	18.7	14.1		
Unemployed	12.5	19.6	20.2	17.4		
Student/pupil	20.8	8.0	6.5	11.8		
Other (livestock keeping)	32.4	44.6	43.1	40.0		
Household income (US$/month)^a^	528.0 (77.9)	201.6 (16.0)	190.3 (15.3)	306.6 (16.5)	84.34	<0.001*^**^
Main source of household income (%)						
Crop sales	0.0	1.8	4.4	2.1	46.26	<0.001*^**^
Sales of livestock	2.2	0.9	2.2	1.8		
Food processing	7.6	0.9	6.7	5.1		
Petty trading	12.0	37.2	36.7	28.6		
Craftsmanship	6.5	0.9	5.6	4.3		
Part‐time labor	17.4	10.6	11.1	13.0		
Permanent employment	47.8	28.3	26.7	34.3		
Pension/remittances	4.3	8.9	2.2	5.1		
Other	2.2	10.6	4.4	5.7		
Household expenditure (%)						
Staple foods and snacks	51.0	33.3	47.1	43.8	41.67	<0.001*^**^
School fee	14.3	14.2	11.5	13.3		
Medical fee	15.3	30.6	25.3	23.7		
Water	4.1	5.6	4.6	4.8		
Transport	1.0	2.7	5.7	3.2		
Accommodation	4.1	7.2	1.1	4.1		
Income spending on foods (%)^a^	45.0 (17.4)	51.6 (14.7)	48.1 (17.8)	48.2 (16.7)	2.93	0.018*
Main source of purchasing foods (%)						
Fresh market	29.9	20.7	9.6	20.1	16.58	0.034*
Supermarket	25.8	16.2	22.3	21.4		
Direct from farm	27.8	45.9	47.9	40.6		
Street	16.5	17.1	20.2	17.9		

Value is the mean (standard deviation).

*Note*. *, **, and *** indicate statistical significance at the 0.1, 0.05, and 0.01 levels, respectively.

Household income and expenditure profiles varied substantially (Table 2). Relating this to household size, the average per capita income was about US$1,039 in Ibanda, US$397 in Kadutu, and US$368 in Bagira. The main source of income in Ibanda was permanent employment (48%), whereas petty trading (37%) was reported as the main source of income in both Kadutu and Bagira. Food was the main item of household expenditure (44%), followed by medical fees (24%), and school fees (13%). The results also found that on average about 48% of households’ income was spent on food. Most respondents in Bagira and Kadutu directly purchased food from farms, while various sources for purchasing food were observed in Ibanda. Although the main source of fuel for cooking was charcoal (86%), more households (16%) in Ibanda had access to electricity than in other communities. About 98% of the main household water supply was from RIGIDESO, the water supply authority in Bukavu.

### 4.2 Household consumption of meat products

In terms of frequency of meat consumption, results showed that beef was the most consumed product, with 83% of the household consuming it at least weekly (Table 3). Goat meat and pork were widely consumed too, with between 66% and 71% of the respondents, respectively, consuming these products weekly. The products least consumed were chicken and rabbit since these are less often produced and available. On average, 68% of the respondents consumed milk in a week, followed by sausage (53%), yogurt (48%), and cheese (45%). Households in Ibanda purchased more fresh meat and meat products than those in Kadutu and Bagira. The average daily consumption in Ibanda was 1.9 times higher for beef than in Kadutu and Bagira, 1.5 times higher for goat meat, 1.5 times higher for pork, and 3.5 times higher for chicken. In the study, it was also found that the price of meat products varied by communities. For example, the price of processed products (sausage, milk, yogurt, and cheese) seemed to be higher in Ibanda than in Kadutu and Bagira.

**Table 3:** Household consumption levels, purchased quantity, and price of meat products

Meat products	Variables	Community			Total	*χ* ^2^	*p*‐value
		Ibanda (*N* = 99)	Kadutu (*N* = 110)	Bagira (*N* = 100)			
Cattle							
Fresh meat	Frequency (%)						
	Daily	23.8	14.1	11.3	16.4	9.59	0.294
	Weekly	65.5	67.7	66.3	66.5		
	Others^a^	10.7	18.2	12.5	17.1		
	Purchased quantity (kg/week)^b^	4.6 (0.4)	2.9 (0.4)	3.7 (0.4)	3.7 (0.4)	4.52	0.018*
	Price (US$/kg)^b^	3.4 (0.1)	3.9 (0.1)	3.2 (0.1)	3.5 (0.1)	12.12	<0.001***
Sausage	Frequency (%)						
	Daily	14.3	10.3	0.0	8.2	16.49	0.086
	Weekly	51.4	24.1	60.0	45.2		
	Others^a^	34.3	65.5	40.0	46.6		
	Purchased quantity (kg/week)^b^	3.8 (1.4)	2.5 (0.8)	2.9 (1.1)	3.1 (1.1)	0.99	0.375
	Price (US$/kg)^b^	4.8 (0.6)	3.5 (0.3)	3.7 (1.1)	4.0 (0.7)	14.31	<0.001***
Milk	Frequency (%)						
	Daily	85.2	24.1	28.6	45.9	43.74	0.368
	Weekly	11.1	13.0	42.9	22.3		
	Others^a^	3.7	62.9	28.6	31.8		
	Purchased quantity (kg/week)^b^	2.7 (0.4)	3.4 (0.4)	4.3 (0.6)	3.5 (0.5)	2.10	0.128
	Price (US$/kg)^b^	1.4 (0.2)	1.2 (0.3)	1.1 (0.1)	1.2 (0.2)	11.44	<0.001***
Yogurt	Frequency (%)						
	Daily	19.1	21.1	6.5	15.6	24.11	0.020*
	Weekly	36.2	31.6	29.0	32.3		
	Others^a^	44.7	47.4	64.5	52.2		
	Purchased quantity (kg/week)^b^	11.9 (4.3)	4.1 (1.0)	4.9 (0.4)	7.0 (1.9)	1.50	0.228
	Price (US$/L)^a^	1.9 (0.2)	1.5 (0.5)	1.6 (0.2)	1.7 (0.3)	2.08	0.130
Cheese	Frequency (%)						
	Daily	15.0	13.0	16.7	14.9	33.91	<0.001***
	Weekly	30.0	25.6	33.3	29.6		
	Others^a^	55.0	60.8	50.0	55.3		
	Purchased quantity (kg/week)^b^	2.2 (0.3)	1.6 (0.2)	2.0 (0.3)	1.9 (0.3)	1.85	0.170
	Price (US$/kg)^b^	4.2 (0.3)	3.4 (0.1)	3.5 (0.4)	3.7 (0.3)	2.44	0.100
Goat							
Fresh meat	Frequency (%)						
	Daily	8.3	5.1	5.8	6.4	4.20	0.838
	Weekly	79.2	60.2	53.0	64.1		
	Others^a^	15.5	34.7	41.3	30.5		
	Purchased quantity (kg/week)^b^	4.2 (0.5)	2.7 (0.3)	2.4 (0.2)	3.1 (0.3)	1.43	0.251
	Price (US$/kg)^b^	3.5 (0.3)	3.8 (0.4)	3.7 (0.4)	3.7 (0.4)	7.10	0.102
Pork							
Fresh meat	Frequency (%)						
	Daily	15.0	13.2	7.0	11.7	13.50	0.197
	Weekly	60.0	57.4	46.5	54.6		
	Others^a^	25.0	32.1	46.5	34.5		
	Purchased quantity (kg/week)^b^	2.4 (0.4)	3.9 (0.6)	3.1 (0.4)	3.1 (0.5)	8.24	<0.001***
	Price (US$/kg)^b^	2.6 (0.1)	3.0 (0.2)	2.4 (0.2)	2.7 (0.2)	2.78	0.065
Chicken							
Alive	Frequency (%)						
	Daily	19.1	6.5	4.5	10.0	55.83	<0.001***
	Weekly	39.7	14.5	14.5	22.9		
	Others^a^	41.2	79.0	80.9	67.1		
	Purchased quantity (kg/week)^b^	4.4 (0.5)	2.8 (0.3)	2.7 (0.2)	3.3 (0.3)	5.38	0.005**
	Price (US$/kg)^b^	2.1 (0.2)	1.9 (0.2)	2.0 (0.2)	2.0 (0.2)	0.99	0.373
Rabbit							
Alive	Frequency (%)						
	Daily	25.0	0.0	0.0	8.3	28.64	0.012
	Weekly	25.0	10.0	12.0	15.7		
	Others^a^	50.0	90.0	88.0	76.0		
	Purchased quantity (kg/week)^b^	2.3 (0.3)	1.5 (0.2)	2.5 (0.3)	2.1 (0.3)	6.95	0.007**
	Price (US$/kg)^b^	3.2 (0.4)	3.4 (0.2)	3.9 (0.3)	3.6 (0.4)	0.09	0.917

Others mean every monthly, 2 months, quarterly, biennially, annually.

Value is the mean (standard deviation).

*, **, and *** indicate statistical significance at the 0.1, 0.05, and 0.01 levels, respectively.

### 4.3 Preference of meat products

Only 47% of the respondents were satisfied with the meat products in the market (Table 4). When asked about the criteria that caused dissatisfaction, 24% claimed unhealthiness and high price as the main criteria, followed by low quantity (18%) and harmful effect (11%). It could be seen that the dissatisfaction can be divided into two groups. The respondents, especially in Kadutu and Bagira, used high price and low quantity as extrinsic criteria; unhealthiness and harmful effect were mainly perceived as intrinsic attributes by the respondents in Ibanda.

**Table 4:** Preference of respondents on all meat products in the market

Variables	Community			Total	*χ* ^*2*^	*p*‐value
	Ibanda (*N* = 99)	Kadutu (*N* = 110)	Bagira (*N* = 100)			
Are you satisfied with the meat products in the market? (%)						
Yes	62.0	46.9	31.9	46.9	17.60	<0.001***
No	38.0	53.1	68.1	53.1		
Which criteria make you dissatisfied with the meat products? (%)						
Less nutritious	14.5	8.5	1.6	8.2	14.33	0.179
Less delicious	11.9	10.2	4.7	8.9		
Unhealthiness	24.3	23.7	25.0	24.3		
Harmful effect	19.3	6.8	7.8	11.3		
Low quantity	9.5	22.0	21.9	17.8		
High price	16.2	23.7	31.3	23.7		
Unavailability	4.3	5.1	7.8	5.7		
Will you accept to pay slightly more for new improved products from meat? (%)						
Yes	66.0	41.6	43.6	50.4	2.13	0.345
No	34.0	58.4	56.4	49.6		
What is the main meat do you like to have its product in the market? (%)						
Beef	46.0	25.8	28.0	33.3	15.52	0.049*
Pork	10.0	30.6	32.0	24.2		
Goat	24.0	16.1	15.0	18.4		
Poultry	10.0	17.7	16.0	14.6		
Rabbit	10.0	9.7	9.0	9.6		

Note. *, **, and *** indicate statistical significance at the 0.1, 0.05, and 0.01 levels, respectively.

When the reason for purchasing new, improved products is considered, the tendency to pay was more in Ibanda (66%) when compared to Kadutu (42%) and Bagira (44%). The result also showed that the respondents demanded more products from beef (40%) compared to pork (21%), goat meat (17%), poultry (15%), and rabbit (8%).

### 4.4 Social factors influencing purchasing decision

Regarding the association between socio‐demographic and socioeconomic factors on purchasing decisions and WTP for meat products, it was observed that living area and gender have a positive significant effect on purchasing decisions but a negative significant effect on WTP (Table 5). Results of the *logit* model also indicate a negative correlation between age and purchasing decisions; a positive correlation was observed between age and WTP.

**Table 5:** Logistic regression for social factors determining consumer purchasing decisions and willingness to pay for meat products

Variables	Purchasing decision		Willingness to pay	
	Estimate	Pr (>|z|)	Estimate	Pr (>|z|)
(Intercept)	−0.228	0.566	−0.268	0.502
Living area	0.761 **	0.003	−0.545 *	0.022
Gender	0.059 *	0.048	0.371*	0.026
Age	0.016*	0.013	0.007*	0.041
Marital status	−0.059	0.737	−0.344	0.054
Low education level (junior/secondary)	0.052	0.791	0.110	0.581
High education level (college/university)	0.120	0.926	0.322	0.140
Employment status of household head	−0.228	0.164	−0.079	0.637
Household size	0.024	0.467	−0.067	0.060
Household with children	−0.001	0.997	0.511	0.057
Household annual income	0.000	0.360	0.000	0.182
AIC	397.6		388.5	
Log likelihood	−187.8		−183.3	
Chi‐square	23.8		19.4	
Chisquare probability	0.008**		0.036*	
Pseudo‐R^2^	0.0596		0.0502	

Note.*, **, and *** indicate statistical significance at the 0.1, 0.05, and 0.01 levels, respectively.

Although other variables were not found to affect purchasing decisions and WTP significantly, when the education level of the household head changes from low to high, the estimated coefficients of purchasing decisions increase by 2.3 times and of WTP by 2.9 times. Marital status and intrahousehold sharing of information were not found to affect purchasing decisions and WTP. Similarly, the employment status of a household head, household size, and the presence of children did not have a significant influence. Surprisingly, household annual income did not play a significant role.

### 4.5 Product attributes influencing purchasing decisions

The results showed that although the respondents were dissatisfied about unhealthiness, harmful effect, high price, and low quantity, these attributes did not exhibit a significant influence on their purchasing decisions and WTP for meat products but color, in‐mouth texture, and availability were identified as significant attributes. The respondents selected availability as the only significant attribute for WTP (Table 6).

**Table 6:** Ordered *probit* regression for product attributes determining consumer purchasing decision and willingness to pay for meat products

Product attributes	Purchasing decision			Willingness to pay		
	Estimate	Odd ratio	*p*‐value	Estimate	Odd ratio	*p*‐value
Nutrition	0.309	1.362	0.059	0.213	1.238	0.188
Color	−0.512	0.599	0.002**	−0.163	0.850	0.314
Texture	−0.399	0.671	0.019*	−0.313	0.731	0.063
Taste	−0.248	0.780	0.134	−0.148	0.863	0.369
Harmful effect	−0.100	0.905	0.553	−0.287	0.751	0.089
Price	0.165	1.180	0.313	0.039	1.040	0.810
Availability	−0.526	0.591	0.002**	−0.459	0.632	0.005**
Quantity	−0.142	0.867	0.389	−0.233	0.792	0.150
Perception	4.277	71.996	0.000***	4.279	72.145	0.000***
Income	0.949	2.582	0.171	0.918	2.504	0.029*

Note. *, **, and *** indicate statistical significance at the 0.1, 0.05, and 0.01 levels, respectively.

## 5 DISCUSSION

Consumers’ preferences, behavior, and perception of meat and meat products depend on many factors, sensory (product‐specific factor), psychological (individual factor), and marketing (environmental factor). These aspects might be altered owing to individual behavior, context, culture, available information (Font‐i‐Furnols & Guerrero, 2014), concerns, lifestyles, and socio‐demographic characteristics (Bernués, Olaizola, & Corcoran, 2003; Grunert et al., 2004). Among socio‐demographic variables, our findings demonstrated that, as expected, living area and gender had a positive significant effect on purchasing decisions but a negative significant effect for WTP. The positive significant effect of living area on purchasing decisions and WTP for meat products indicated that people living in rural areas make a decision to purchase meat products differently from those living in urban areas. While a higher rate of WTP among respondents for meat products was found in urban areas, price alone cannot be used to infer the actual WTP of respondents because they were aware of the artificial purchase situation. Consumers often claim that they would pay higher prices for certain product attributes than they actually do in real purchase situations (Feldmann & Hamm, 2015). For gender effect, Croson and Gneezy (2009) stated that men and women apparently vary in their emotional response to uncertain situations and this difference results in dissimilarities in risk taking. In food purchasing, women are more selective and tend to integrate multiple cues in the household more than men. In contrast, men are generally more confident and more willing to take risks in purchasing complex products/services than women (Erasmus, Donoghue, & Dobbelstein, 2014). Cavaliere, Ricci, and Banterle (2015) reported that women are more concerned about a healthy diet and have high levels of personal knowledge on food characteristics, and thus, they are more careful than men about what they eat. Dibb and Fitzpatrick (2014) also showed that men tend to consume more meat than women and are less willing to consider reducing their consumption.

A negative correlation between age and purchasing decision suggests that younger people were less concerned in making decisions to purchase than older people. In contrast, a positive correlation between age and WTP shows that older and more experienced people tend to be more conscious about the meat products they buy. Although household income did not play a significant role in this study, pork and poultry products were mostly demanded by respondents from Kadutu and Bagira, while those from Ibanda rated beef and goat meat highly. This result can be explained by the fact that beef and goat are sold in large portions that require refrigeration: Pork and poultry are mostly sold in smaller portions that do not need it. People in Ibanda who have access to more electricity are likely to purchase and consume goat meat. Likewise, higher income and more educated consumers in Ibanda may prefer quality rather than quantity of products when compared to consumers in Kadutu and Bagira. This could be explained by the budget constraints of lower‐income households that may be limited to cheaper choices (Morales & Higuchi, 2018). This is in agreement with the findings of Jolly, Bayard, Awuah, Fialor, and Williams (2009) and Sabran, Jamaluddin, Abdul Mutalib, and Abdul Rahman (2012) who mentioned that wealthier consumers are more likely to take precautions about food and are more willing to pay for high‐quality products than those with lower incomes. Additionally, Silva, Caro, and Magana‐Lemus (2016) also found that food‐secure households with higher incomes purchase a wider variety of high‐quality food items than food‐insecure households with lower incomes. However, this finding contrasts with the studies reported by Robert, Manolis, and Tanner (2003) who reported that lower‐income consumers are more concerned about the value of money and with not wasting their money on goods and services that do not meet their basic needs (Erasmus et al., 2014).

Moreover, it could be seen that the more educated people in Ibanda generally have higher incomes; thus, they might have more options than less educated people when purchasing meat products. Also, people in Ibanda might be sensitive to quality since meat products can be a risk factor for their health. In Ibanda, high blood pressure (57%), high cholesterol levels (21%), and incidence of diabetes (20%) were reported as a cause of specific dietary requirements, while in Kadutu and Bagira, the averages reported were 41% for high blood pressure, 16% for high cholesterol, and 14% for diabetes (data not shown). A study by Chen, Anders, and An (2013) showed that consumer willingness to purchase also increased with level of education; and the education level was positively linked to consumers’ willingness to adopt new products (Huotilainen, Pirttila‐Backman, & Tuorila, 2006). However, these results are opposed to those of Dellaert, Arentze, and Timmermans (2008) who reported that less educated consumers might lack the cognitive ability to comprehend the implications of their purchasing decisions and might subsequently not be bothered about all functional and quality‐related issues compared with more educated consumers. The meat consumption trends in Eastern DRC seem differ from the European trend. For example, Germans with higher education are more likely to consume less meat or follow a vegetarian diet than lower educated people (Pfeiler & Egloff, 2018).

Luning, Marcelis, and Jongen (2002) mentioned that quality represents the features/properties of a product that result in satisfying the consumers’ physiological and/or psychological needs. Dransfield (2005) also suggested that at least two attributes of appearance are normally used by consumers in quality judgements on meat. For instance, cut type, color, and fat structure and levels have been observed as influential in calculating quality expectations (Grunert et al., 2004). When the influence of product attributes on purchasing decisions in this study is considered, quality aspects such as color and in‐mouth texture cannot be ignored. Color as an intrinsic quality attribute influences consumers’ expectations of meat quality at the moment of purchase (Carpenter, Cornforth, & Whittier, 2001; Font‐i‐Furnols & Guerrero, 2014; Gracia & de Magistris, 2013; Verbeke et al., 2005; West, Larue, Touil, & Scott, 2001), probably because consumers normally use color to indicate wholesomeness or contamination of meat products (Mancini, 2009; Owusu‐Sekyere et al., 2014). On the other hand, eating quality and in‐mouth texture are found to be highly correlated with the overall experienced quality, attitude to purchase, and WTP for meat products (Lusk et al., 2001; Banović, Grunert, Barreira, & Aguiar Fontes, 2009). Robbins et al. (2003) reported consumers were most concerned with color, fat content, price, and type of cut when purchasing beef, whereas texture and flavor were most important in determining eating satisfaction.

**Figure 3:**
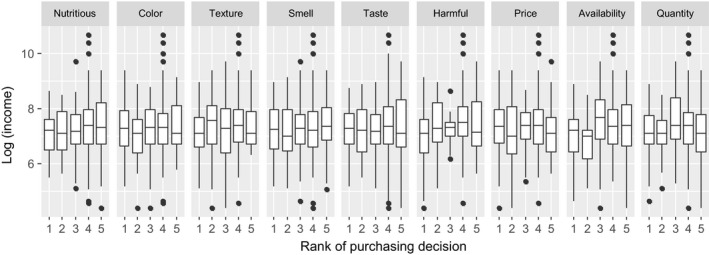
Influence of product attributes on consumers’ purchasing decision according to the household income fluctuation

**Figure 4:**
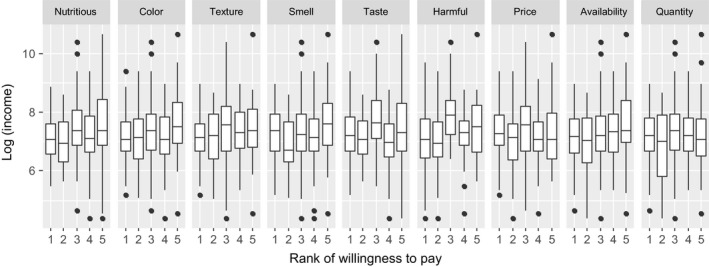
Influence of product attributes on WTP according to the household income fluctuation

The findings from this study also suggest that availability (marketing factor) is one of the most important attributes that influences purchasing decisions and WTP of meat products. Availability is one reason that can explain, for instance, the lack of access to markets and market information that had a negative influence on consumers’ WTP and purchase behavior toward food products (Zundel and Kilcher, 2007; Young, Hwang, McDonald, & Oates, 2010). Young et al. (2010) also mentioned that consumers generally do not like to spend much time searching for food products although perception, a psychological motivator for purchasing meat products, affects the process for consumers in selecting, organizing, and interpreting information related to meat products (Kotler, Armstrong, Harris, & Piercy, 2013). This factor is important in shaping consumers’ acceptance, purchase, and future consumption, as stated by Grunert, Verbeke, Kügler, Saeed, and Scholderer (2011). The results in this study exhibited a significant effect on consumers’ perception in both purchasing decisions and WTP. Although income was not significant in the linear regression model, it was found that income played a significant role in WTP as analyzed by ordered multinomial logistic regression. From the household income results (gg plot as presented in Figures 3,4), it appears that the higher the income, the better the consideration that is given to nutrition, harmful effect, and availability as important factors on purchasing decisions and WTP. This result agrees with the findings of Henchion, McCarthy, Resconi, and Troy (2014) who pointed out that the influence of factors such as income and price are likely to decline over time so that other factors, such as quality, will become more important in purchasing meat products.

## 6 CONCLUSIONS

This exploratory study investigated the preference and WTP for meat products of Congolese consumers in Eastern DRC. The study revealed that women and older consumers from urban areas were more likely to purchase meat products. Although the respondents were expected/hypothesized to rate healthiness, quantity, and the low price of products, consumers’ decisions to purchase meat products are more often based on sensory factors such as color and in‐mouth texture as well as on marketing factors such as availability. Availability played a prominent/key role on their WTP. However, nutrition, harmful effect, and availability tended to be taken into consideration in higher income groups. This result is related to personal WTP and is a consequence of consumers’ poor access to information about meat quality. Therefore, public efforts are needed to address knowledge gaps through awareness campaigns that promote and disseminate information about meat quality. In summary, the empirical findings presented here reveal new and essential insights into consumers’ preferences and their purchase of meat products (in a region where food insecurity is prevalent). These insights provide practical insights for actors in the meat value chain to better satisfy consumers’ expectations, demands, and needs. The findings can be used to identify opportunities for livestock farmers to commercialize livestock enterprises for income and employment generation, thus contributing to improving nutrition and alleviating poverty. These insights can also be of relevance to countries with similar socioeconomic characteristics in low‐income countries.

## CONFLICT OF INTEREST

The authors have no conflict of interests.

## ETHICAL STATEMENT

This study does not involve any human or animal testing.

## References

[fsn3813-bib-0001] Agresti, A. (2002). Categorical data analysis. Second (p. 710). Hoboken, New Jersey: John Wiley & Sons, Inc 10.1002/0471249688

[fsn3813-bib-0002] Akshita, J. , Ramyani, M. , Sridevi, T. , & Trishita, B. (2013). Multinomial logit models. Econometrics paper terms.

[fsn3813-bib-0003] Alemu, M. H. , Olsen, S. B. , Vedel, S. E. , Pambo, K. O. , & Owino, V. O. (2017). Combining product attributes with recommendation and shopping location attributes to assess consumer preferences for insect‐based food products. Food Quality and Preference, 55, 45–57. 10.1016/j.foodqual.2016.08.009

[fsn3813-bib-0004] Banović, M. , Grunert, K. G. , Barreira, M. M. , & Aguiar Fontes, M. (2009). Beef perception at the point of purchase: A study from Portugal. Food Quality and Preference, 20, 335–342. 10.1016/j.foodqual.2009.02.009

[fsn3813-bib-0005] Bernués, A. , Olaizola, A. , & Corcoran, K. (2003). Labelling information demanded by European consumers and relationships with purchasing motives, quality and safety of meat. Meat Science, 65, 1095–1106. 10.1016/S0309-1740(02)00327-3 22063692

[fsn3813-bib-0006] Box, G. E. P. , & CoX, D. R. (1964). An analysis of transformations. Journal of the Royal Statistical Society, Series B., 26, 211–246.

[fsn3813-bib-0007] Carpenter, C. E. , Cornforth, D. P. , & Whittier, D. (2001). Consumer preferences for beef color and packaging did not affect eating satisfaction. Meat Science, 57, 359–363. 10.1016/S0309-1740(00)00111-X 22061707

[fsn3813-bib-0008] Cavaliere, A. , Ricci, E. C. , & Banterle, A. (2015). Nutrition and health claims: Who is interested? An empirical analysis of consumer preference in Italy. Food Quality and Preference, 41, 44–51. 10.1016/j.foodqual.2014.11.002

[fsn3813-bib-0009] Chen, Q. , Anders, S. , & An, H. (2013). Measuring consumer resistance to a new food technology: A choice experiment in meat packaging. Food Quality and Preference, 28, 419–428. 10.1016/j.foodqual.2012.10.008

[fsn3813-bib-0010] Croson, R. , & Gneezy, U. (2009). Gender differences in preferences. Journal of Economic Literature, 47(2), 448–474. 10.1257/jel.47.2.448

[fsn3813-bib-0011] Delgado, C. L. (2003). Rising consumption of meat and milk in developing countries has created a new food revolution. Journal of Nutrition, 133(11), 3907S–3910S. 10.1093/jn/133.11.3907S 14672289

[fsn3813-bib-0012] Dellaert, B. G. C. , Arentze, T. A. , & Timmermans, H. J. P. (2008). Shopping context and consumers’ mental representation of complex shopping trip decision problems. Journal of Retailing, 84(2), 219–232. 10.1016/j.jretai.2008.02.001

[fsn3813-bib-0013] Dibb, S. , & Fitzpatrick, I. (2014). Let's talk about meat: Changing dietary behaviour for the 21st century. London, UK: Eating Better.

[fsn3813-bib-0014] Dodge, Y. (2008). The Concise Encyclopedia of Statistics.(p. 616). New York, USA: Springer‐Verlag.

[fsn3813-bib-0015] Dransfield, E. (2005). Consumer importance in creating demands for meat and meat product safety. Technologija Mesa, 46(1–2), 3–10.

[fsn3813-bib-0016] Engel, J. (1988). Polytomous Logistic Regression. Statistica Neerlandica, 42, 233‐252. 10.1111/j.1467-9574.1988.tb01238.x.

[fsn3813-bib-0017] Erasmus, A. C. , Donoghue, S. , & Dobbelstein, T. (2014). Consumer's perception of the complexity of selected household purchase decisions. Journal of Retailing and Consumer Services, 21, 293–305. 10.1016/j.jretconser.2014.02.008

[fsn3813-bib-0018] FAO Statistics (FAOSTAT) . (2018). Rome, Italy.

[fsn3813-bib-0019] Faraway, J. (2006). Extending the linear model with R. generalized linear, mixed effects and nonparametric regression models (p. 322). New York, NY: Taylor & Francis Group.

[fsn3813-bib-0020] Feldmann, C. , & Hamm, U. (2015). Consumer's perceptions and preferences for local food: A review. Food Quality and Preference, 40(Part A), 152–164. 10.1016/j.foodqual.2014.09.014

[fsn3813-bib-0021] Fiala, N. (2008). Meeting the demand: An estimation of potential future greenhouse gas emissions from meat production. Ecological Economics, 67(3), 412–419. 10.1016/j.ecolecon.2007.12.021

[fsn3813-bib-0022] Font‐i‐Furnols, M. , & Guerrero, L. (2014). Consumer preference, behavior and perception about meat and meat products: An overview. Meat Science, 98, 361–371. 10.1016/j.meatsci.2014.06.025 25017317

[fsn3813-bib-0023] Food and Agriculture Organization (FAO) (2005). Livestock sector brief – Congo. Rome, Italy: FAO.

[fsn3813-bib-0024] Food and Agriculture Organization (FAO) (2011). World livestock 2011 – Livestock in food security. Rome, Italy: FAO.

[fsn3813-bib-0025] Food and Agriculture Organization (FAO) (2013). The state of food and agriculture 2013. Rome, Italy: FAO.

[fsn3813-bib-0026] Food and Agriculture Organization (FAO) (2014). Meat and meat product. Rome, Italy: Food Outlook: Biannual report on global food markets.

[fsn3813-bib-0027] Fox, J. (2008). Applied regression analysis and generalized linear models, 2nd ed Thousand oaks, California, USA: Sage.

[fsn3813-bib-0028] Gracia, A. , & de Magistris, T. (2013). Preferences for lamb meat: A choice experiment for Spanish consumers. Meat Science, 95, 396–402. 10.1016/j.meatsci.2013.05.006 23747635

[fsn3813-bib-0029] Grunert, K. G. , Bredahl, L. , & Brunsø, K. (2004). Consumer perception of meat quality and implications for product development in the meat sector‐a review. Meat Science, 66, 259–272. 10.1016/S0309-1740(03)00130-X 22064127

[fsn3813-bib-0030] Grunert, K. G. , Verbeke, W. , Kügler, J. O. , Saeed, F. , & Scholderer, J. (2011). Use of consumer insight in the new product development process in the meat sector. Meat Science, 89, 251–258. 10.1016/j.meatsci.2011.04.024 21605939

[fsn3813-bib-0031] Henchion, M. , McCarthy, M. , Resconi, V. C. , & Troy, D. (2014). Meat consumption: Trends and quality matters. Meat Science, 98, 561–568. 10.1016/j.meatsci.2014.06.007 25060586

[fsn3813-bib-0032] Hoddinott, J. (2016). The economics of reducing malnutrition in sub‐Saharan Africa. Global panel on agriculture and food systems for nutrition working paper. Retrieved from https://glopan.org/sites/default/files/Global_Panel_Working_Paper.pdf (accessed on 24.8.2018).

[fsn3813-bib-0033] Howard, P. H. , & Allen, P. (2008). Consumer willingness to pay for domestic “fair trade”: evidence from the United States. Renewable Agriculture and Food System, 23(3), 235–242. 10.1017/S1742170508002275

[fsn3813-bib-0034] Hung, Y. , de Kok, T. M. , & Verbeke, W. (2016). Consumer attitude and purchase willingness towards processes meat products with natural compounds and a reduced level of nitrite. Meat Science, 121, 119–126. 10.1016/j.meatsci.2016.06.002 27310600

[fsn3813-bib-0035] Huotilainen, A. , Pirttila‐Backman, A. M. , & Tuorila, H. (2006). How innovativeness relates to social representation of new foods and to the willingness to try and use such foods. Food Quality and Preference, 17(5), 353–361. 10.1016/j.foodqual.2005.04.005

[fsn3813-bib-0036] Jolly, C. M. , Bayard, B. , Awuah, R. T. , Fialor, S. C. , & Williams, J. T. (2009). Examining the structure of awareness and perceptions of groundnut aflatoxin among Ghanaian health and agricultural professionals and its influence on their actions. The Journal of Socio‐Economics, 38, 280–287. 10.1016/j.socec.2008.05.013

[fsn3813-bib-0037] Kandala, N. B. , Madungu, T. P. , Emina, J. B. O. , Nzita, K. P. D. , & Cappuccio, F. P. (2011). Malnutrition among children under the age of five in the Democratic Republic of Congo (DRC): Does geographic location matter? BMC Public Health, 11(261), 15 (cited 2017 September 10), Retrieved from http://www.biomedcentral.com/1471-2458/11/261/abstract/. 21518428 10.1186/1471-2458-11-261 PMC3111378

[fsn3813-bib-0038] Kanerva, M. (2013). Meat consumption in Europe: Issues, trends and debates. Artect‐paper, 187. January. Germany: Universität Bremen.

[fsn3813-bib-0039] Kotler, P. , Armstrong, G. , Harris, L. C. , & Piercy, N. (2013). Principles of marketing(6th, European ed.) Harlow: Pearson Education.

[fsn3813-bib-0040] KPMG (2016). Economic snapshot H2, 2016 – Democratic Republic of Congo. Retrieved from https://home.kpmg.com/content/dam/kpmg/za/pdf/2016/10/KPMG-DRC-2016-Snapshot.pdf. (accessed 27.8.2018).

[fsn3813-bib-0041] Luning, P. , Marcelis, W. , & Jongen, W. (2002). Food quality management. A technico‐managerial approach. Wageningen, The Netherlands: Wageningen Press.

[fsn3813-bib-0501] Lusk, J. L. , Fox, J. A. , Schroeder, T. C. , Mintert, J. , & Koohmaraie, M. (2001). In‐Store Valuation of Steak Tenderness. American Journal of Agricultural Economics, 83, 539–550.

[fsn3813-bib-0042] Maass, B. L. , Musale, D. K. , Chiuri, W. L. , Gassner, A. , & Peters, M. (2012). Challenges and opportunities for smallholder livestock production in post‐conflict South Kivu, eastern DR Congo. Tropical Animal Health and Production, 44, 1221–1232. 10.1007/s11250-011-0061-5 22286398 PMC3382655

[fsn3813-bib-0043] Mahangaiko, M. , Mabi, N. , Bakana, M. , & Nyonggombe, U. (2015). Food contamination with salmonella and human health in Kinshasa city, Democratic Republic of Congo (DRC). Journal of Applied Bioscience, 94, 8809–8814. 10.4314/jab.v94i1.3

[fsn3813-bib-0044] Makelele, L. K. , Kazadi, Z. A. , Oleko, R. W. , Foma, R. , Mpalang, R. K. , Ngbolua, K. N. N. , & Gedeon, B. (2015). Microbiological quality of food sold by street vendors in Kisangani, Democratic Republic of Congo. African Journal of Food Science, 9(5), 285–290.

[fsn3813-bib-0045] Mancini, R. A. (2009). Meat color In Kerry J. R., & Ledward D. (Eds.), Improving the sensory and nutritional quality of fresh meat (pp. 89–110). Cambridge, England: CRC Press: Woodhead Publishing Limited 10.1533/9781845695439.1.89

[fsn3813-bib-0046] Menard, S. (2002). Applied logistic regression analysis (p. 91). Thousand oaks, California, USA: SAGE 10.4135/9781412983433

[fsn3813-bib-0047] Mockshell, J. , Ilukor, J. , & Birner, R. (2014). Providing animal health services to the poor in Northern Ghana: Rethinking the role of community animal health workers? Tropical Animal Health and Production, 46(2), 475–480. 10.1007/s11250-013-0518-9 24346862

[fsn3813-bib-0048] Morales, L. E. , & Higuchi, A. (2018). Is fish worth more than meat? – How consumers’ beliefs about health and nutrition affect their willingness to pay more for fish than meat. Food Quality and Preference, 65, 101–109. 10.1016/j.foodqual.2017.11.004

[fsn3813-bib-0049] Neumann, C. G. , Bwibo, N. O. , Murphy, S. P. , Sigman, M. , Whaley, S. , Allen, L. H. , … Demment, M. W. (2003). Animal source foods improve dietary quality, micronutrient status, growth and cognitive function in Kenya school children: Background, study design and baseline findings. The Journal of Nutrition, 133(11), 3941S–3949S. 10.1093/jn/133.11.3941S 14672294

[fsn3813-bib-0050] Niyonzima, E. , Ongold, M. P. , Brostaux, Y. , Koulagenko, N. K. , Daube, G. , Kimonyo, A. , & Sindic, M. (2017). Consumption patterns, bacteriological quality and risk factors for Salmonella contamination in meat‐based meals consumed outside the home in Kigali, Rwanda. Food Control, 73(Part B), 546–554. 10.1016/j.foodcont.2016.09.004

[fsn3813-bib-0051] Owusu‐Sekyere, E. , Owusu, V. , & Jordaan, H. (2014). Consumer preferences and willingness to pay for beef food safety assurance labels in the Kumasi Metropolis and Sunyani Municipality of Ghana. Food Control, 46, 152–159. 10.1016/j.foodcont.2014.05.019

[fsn3813-bib-0052] Pereira, P. M. D. C. C. , & Vicente, A. F. D. R. B. (2013). Meat nutritional composition and nutritive role in the human diet. Meat Science, 93(3), 586–592. 10.1016/j.meatsci.2012.09.018 23273468

[fsn3813-bib-0053] Pfeiler, T. M. , & Egloff, B. (2018). Personality and attitudinal correlates of meat consumption: Results of two representative German samples. Appetite, 121, 294–301. 10.1016/j.appet.2017.11.098 29154886

[fsn3813-bib-0055] Popkin, B. M. , Adair, L. S. , & Ng, S. W. (2012). Global nutrition transition and the pandemic of obesity in developing countries. Nutrition Review, 70(1), 3–21. 10.1111/j.1753-4887.2011.00456.x PMC3257829 22221213

[fsn3813-bib-0502] R Development Core Team . (2015). R Foundation for Statistical Computing, Vienna, Austria.

[fsn3813-bib-0056] Randolph, T. F. , Schelling, E. , Grace, D. , Nicholson, C. F. , Leroy, J. L. , Cole, D. C. , … Ruel, M. (2007). Invited Review: Role of livestock in human nutrition and health for poverty reduction in developing countries. Journal of Animal Science, 85(11), 2788–2800. 10.2527/jas.2007-0467 17911229

[fsn3813-bib-0057] Reicks, A. L. , Brooks, J. C. , Garmyn, A. J. , Thompson, L. D. , Lyford, C. L. , & Miller, M. F. (2011). Demographics and beef preferences affect consumer motivation for purchasing fresh beef steaks and roasts. Meat Science, 87, 403–411. 10.1016/j.meatsci.2010.11.018 21159449

[fsn3813-bib-0058] Robbins, K. , Jensen, J. , Ryan, K. J. , Homco‐Ryan, C. , McKeith, F. K. , & Brewer, M. S. (2003). Consumer attitudes towards beef and acceptability of enhanced beef. Meat Science, 65, 721–729. 10.1016/S0309-1740(02)00274-7 22063433

[fsn3813-bib-0059] Robert, J. A. , Manolis, C. , & Tanner, J. F. (2003). Family structure, materialism and compulsive buying: A reinquiry and extension. Journal of the Academy of Marketing Science, 31(3), 300–312. 10.1177/0092070303031003007

[fsn3813-bib-0060] Rossi, L. , Hoerz, T. , Thouvenot, V. , Pastore, G. , & Michael, M. (2006). Evaluation of health, nutrition and food security programmes in a complex emergency: The case of Congo as an example of a chronic post‐conflict situation. Public Health Nutrition, 9(5), 551–556. 16923285 10.1079/phn2005928

[fsn3813-bib-0061] Sabran, M. R. , Jamaluddin, R. , Abdul Mutalib, M. S. , & Abdul Rahman, N. (2012). Socio‐demographic and socio‐economic determinants of adults’ knowledge on fungal and aflatoxin contamination in the diets. Asian Pacific Journal of Tropical Biomedicine, 2(3), S1835–S1841. 10.1016/S2221-1691(12)60504-8

[fsn3813-bib-0062] Schumacher, T. , Schroeder, T. C. , & Tonsor, G. T. (2012). Willingness‐to‐pay for calf health programs and certification agents. Journal of Agricultural and Applied Economics, 44(2), 191–202. 10.1017/S1074070800000262

[fsn3813-bib-0063] Shan, L. C. , Henchion, M. , De Brún, A. , Murrin, C. , Wall, P. G. , & Monahan, F. J. (2017). Factors that predict consumer acceptance of enriched processed meats. Meat Science, 133, 185–193. 10.1016/j.meatsci.2017.07.006 28711465

[fsn3813-bib-0064] Silva, A. , Caro, J. C. , & Magana‐Lemus, D. (2016). Household food security: Perceptions, behavior and nutritional quality of food purchases. Journal of Economic Psychology, 55, 139–148. 10.1016/j.joep.2016.05.003

[fsn3813-bib-0503] Tonsor, G. T. , Schroeder, T. C. , Fox, J. A. , & Biere, A. (2005). European preferences for beef steak attributes. Journal of Agricultural and Resource Economics, 103, 367–380.

[fsn3813-bib-0065] United Nations Office for the Coordination of Humanitarian Affairs (OCHA) (2016). DRC strategic response plan 2016. Geneva, Switzerland: Humanitarian needs overview‐DRC https://www.unocha.org/sites/dms/Documents/OCHAin2016.pdf (accessed 25.8.2018).

[fsn3813-bib-0066] Van Acker, F. (2005). Where did all the land go? Enclosure and social struggle in Kivu (DR Congo). Review of African Political Economy, 103, 79–98. 10.1080/03056240500120984

[fsn3813-bib-0067] Van Wezemael, L. , Verbeke, W. , de Barcellos, M. D. , Scholderer, J. , & Perez‐Cueto, F. (2010). Consumer perceptions of beef healthiness: Results from a qualitative study in four European countries. BMC Public Health, 10, 342 10.1186/1471-2458-10-342 20550647 PMC2893462

[fsn3813-bib-0068] Verbeke, W. , De Smet, S. , Vackier, I. , Van Oeckel, M. J. , Warnants, N. , & Van Kenhove, P. (2005). Role of intrinsic search cues in the formation of consumer preferences and choice for pork chops. Meat Science, 69, 343–354. 10.1016/j.meatsci.2004.08.005 22062827

[fsn3813-bib-0069] Vlassenroot, K. , Ntububa, S. , & Raeymaekers, T. (2003). Food security responses to the protracted crisis context of the Democratic Republic of the Congo, paper presented at International workshop on food security in complex emergencies: Building policy frameworks to address longer‐term programming in complex emergencies, 23‐25 Sep. 2003, Tivoli, Italy, (Food and Agriculture Organization of the United Nations (FAO/ESA)), (cited 2017 September 10), Retrieved from http://www.foodsec.org/docs/DRC_overview_web.pdf.

[fsn3813-bib-0070] West, G. E. , Larue, B. , Touil, C. , & Scott, S. L. (2001). The perceived importance of veal meat attributes in consumer choice decisions. Agribusiness, 17, 365–382. 10.1002/(ISSN)1520-6297

[fsn3813-bib-0071] Yamaguchi, R. (2015). Food consumption and preferences of the Bongando people in the Democratic Republic of the Congo. African Study Monographs, 51, 37–55.

[fsn3813-bib-0072] York, R. , & Gossards, M. (2004). Cross‐national meat and fish consumption: Exploring the effects of modernisation and ecological context. Ecological Economics, 48, 239–302.

[fsn3813-bib-0073] Young, W. , Hwang, K. , McDonald, S. , & Oates, C. J. (2010). Sustainable consumption: Green consumer behaviour when purchasing products. Sustainable Development, 18(1), 20–31.

[fsn3813-bib-0074] Zimmerman, L. C. , Schroeder, T. C. , Dhuyvetter, K. C. , Olson, K. C. , Stokka, G. L. , Seeger, J. T. , Grotelueschen, D. M. (2012). The effect of value‐added management on calf prices at superior livestock auction video markets. Journal of Agricultural and Resource Economics, 37(1), 128–143.

[fsn3813-bib-0075] Zozo, R. , Paul, B. K. , Maass, B. L. , Muhimuzu, F. , Bacigale, S. , & Chiuri, W. L. (2012). Value chain assessment of monogastric animal products in Sud‐Kivu, DR Congo. Working report, International Center for Tropical Agriculture (CIAT), Nairobi, Kenya.

[fsn3813-bib-0504] Zundel, C. , & Kilcher, L . (2007). Issues Paper: Organic agriculture and food availabilty. International Conference on Organic Agriculture and Food Security, Rome, 3‐5 May 2007. http://www.fao.org/ORGANICAG/ofs/index_en.htm

